# Supplementary material for: Evidence of a distinct collective mode in Kagome superconductors
Source: Nat Commun. 2024 Jul 19;15:6109. doi: 10.1038/s41467-024-50330-z (PMC11271580; doi:10.1038/s41467-024-50330-z)
Supplement: Supplementary file 1 — Supplementary Information [file 41467_2024_50330_MOESM1_ESM.pdf]

Supplementary Information for

Evidence of a distinct collective mode in kagome superconductors

Bin Hu[1,2#], Hui Chen[1,2,3 #], Yuhan Ye[1,2#], Zihao Huang[1,2], Xianghe Han[1,2], Zhen Zhao[1,2], Hongqin Xiao[1,2], Xiao Lin[1,2], Haitao Yang[1,2], Ziqiang Wang[4], and Hong-Jun Gao[1,2,3*]*

[1] *Beijing National Center for Condensed Matter Physics and Institute of Physics, Chinese Academy of Sciences, Beijing 100190, PR China*

[2] *School of Physical Sciences, University of Chinese Academy of Sciences, Beijing 100190, PR China*

[3]*Hefei National Laboratory, 230088 Hefei, Anhui, PR China*

[4]*Department of Physics, Boston College, Chestnut Hill, MA 02467, USA*

[#]These authors contributed equally to this work
[*]Correspondence to: wangzi@bc.edu, hjgao@iphy.ac.cn

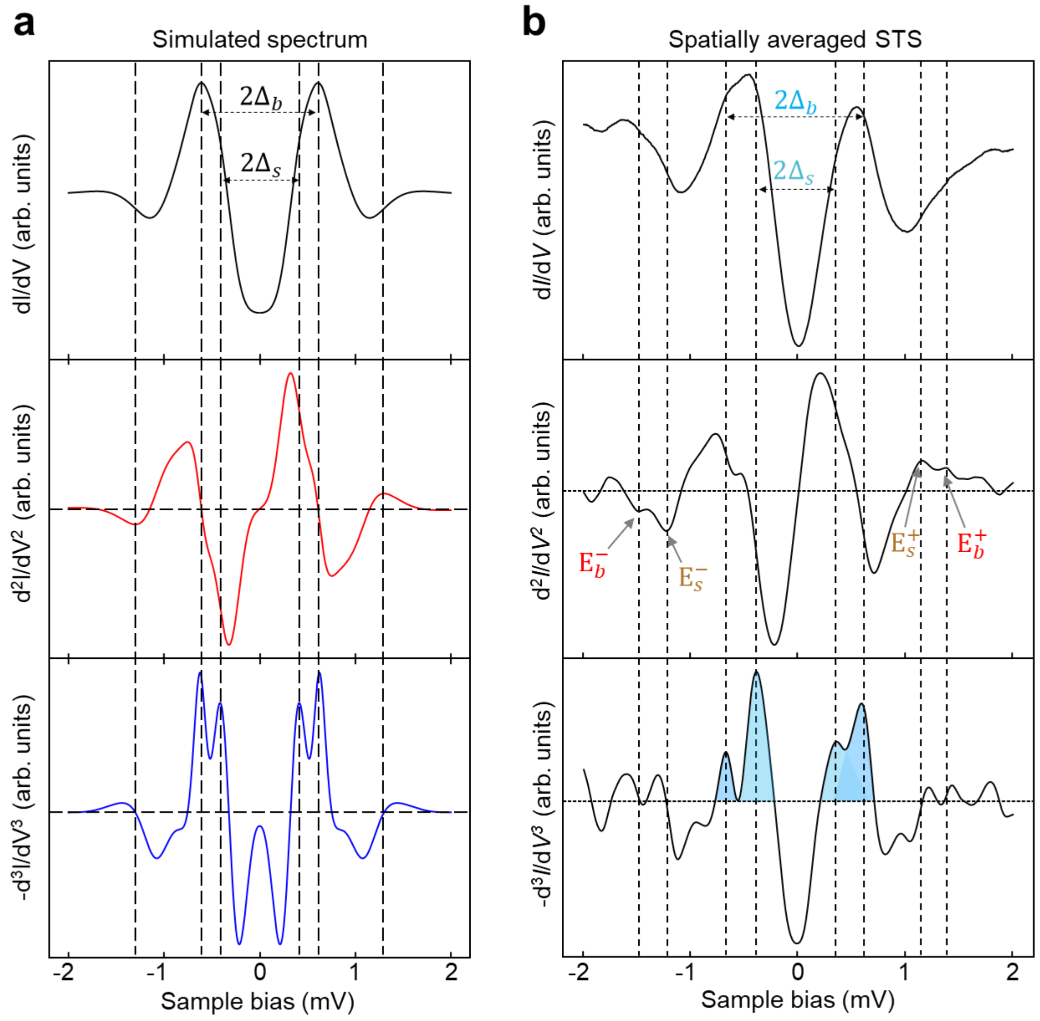

Supplementary Figure 1. Analysis of two-SC gaps and bosonic mode based in pristine CsV_3Sb_5. a, Simulated differential conductance spectrum (dI/dV), showing the dip-peak-hump features (upper panel). The corresponding derivative spectrum (d^2I/dV^2), showing a dip-peak pair corresponding to the maximum slope from dip to hump (middle panel). The negative second derivative spectrum (-d^3I/dV^3), showing the magnified peaks in the dI/dV spectrum (lower panel). **b**, Spatially-averaged dI/dV spectrum mainly from the region in Fig. 1**c** and the corresponding d^2I/dV^2 and -d^3I/dV^3 spectra, showing two SC gaps accompanied by a pair of peak-dip-hump features just outside the SC gaps (V_s=-2.0 mV, I_t=1 nA, V_{mod}=0.05 mV). The two SC peaks are marked by the colored shadow region in -d^3I/dV^3 (lower panel). The vertical dashed lines in **a** and **b** present the peaks/dips, and the horizontal dashed lines present the zero values in d^2I/dV^2 and -d^3I/dV^3.

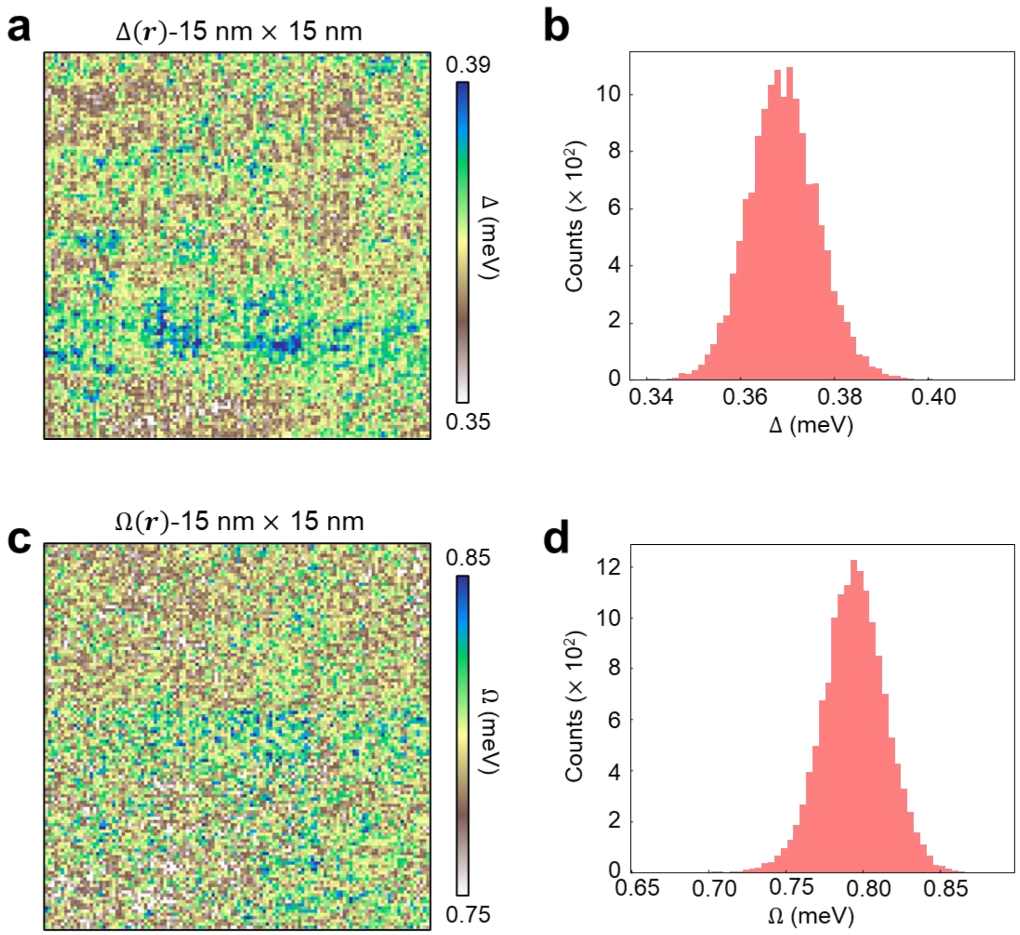

Supplementary Figure 2. Spatial distribution of $\Delta(\boldsymbol{r})$ and $\Omega(\boldsymbol{r})$ of CsV_3Sb_5. a, Spatial distribution of $\Delta(\boldsymbol{r})$ obtained on the Sb surface within the field of view of 15 nm × 15 nm, showing the spatially inhomogeneity of superconducting gap (V_s=-2.0 mV, I_t=1 nA, V_{mod}=0.1 mV). **b**, The histogram plot of **a**. **c**, Spatial distribution of $\Omega(\boldsymbol{r})$ obtained on the same field of view of **a**, showing the spatial inhomogeneity of the bosonic mode. **d**, The histogram plot of **c**.

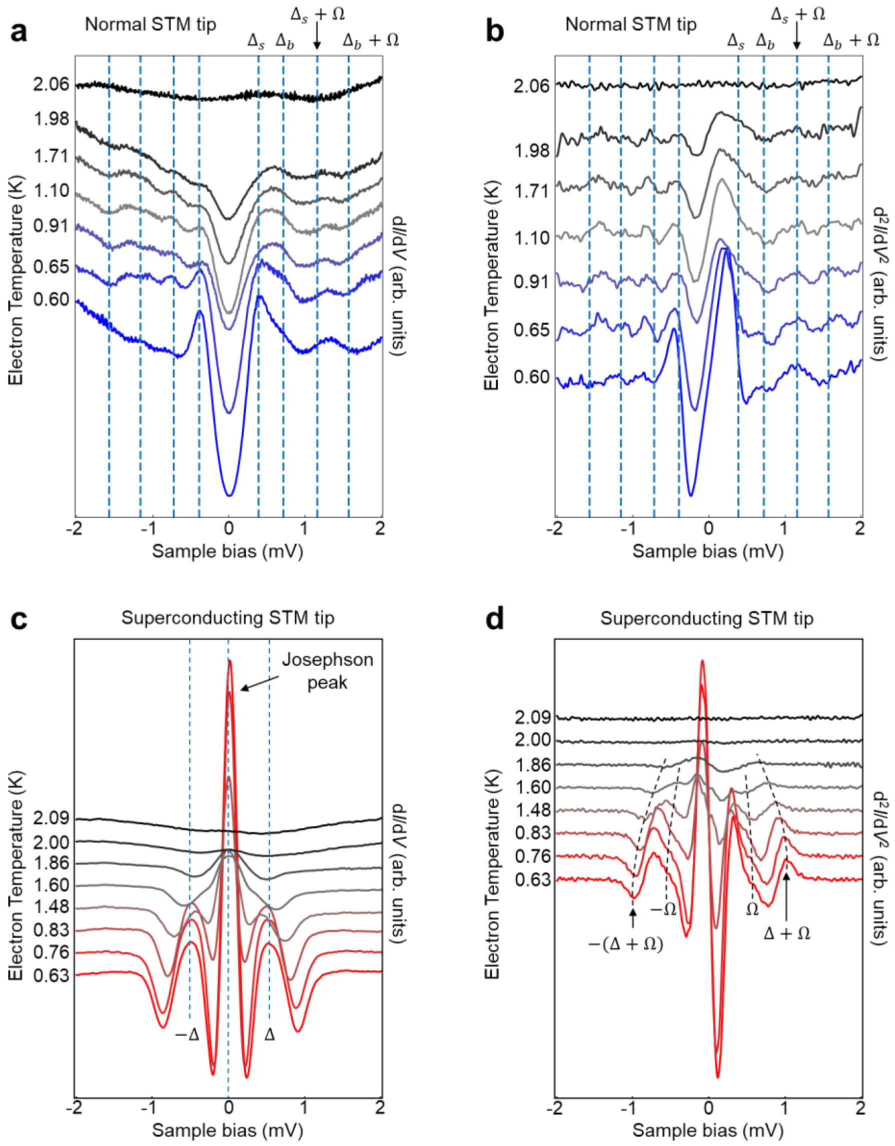

Supplementary Figure 3. Temperature-dependent d*I*/d*V* (d^2I/dV^2) spectra obtained using the normal STM tip and the superconducting STM tip. **a**, A series of d*I*/d*V* spectra obtained by normal STM tip under different temperatures, showing the superconducting gaps and the bosonic mode gradually disappear with increasing temperature, and are invisible when electron temperature $T_{electron}$>2.06 K (V_s=-2.0 mV, V_{mod}=0.1 mV, I_t=1 nA). **b**, A series of derivative spectra (d^2I/dV^2) obtained from **a**. **c**, A series of d*I*/d*V* spectra obtained by superconducting STM tip under different temperatures, showing clearly the signal of Josephson peak, superconducting coherence peak, and bosonic mode, and those signals are weakening with increasing temperature, giving a phase transition temperature of $T_{electron}$~2.00 K (V_s=-2.0 mV, V_{mod}=0.1 mV, I_t=600 nA**)**. **d**, A series of derivative spectra obtained from **c**. Superconducting peaks, bosonic mode, and the Josephson peak in **a**, **b**, **c**, and **d** are labeled and marked by the dashed lines.

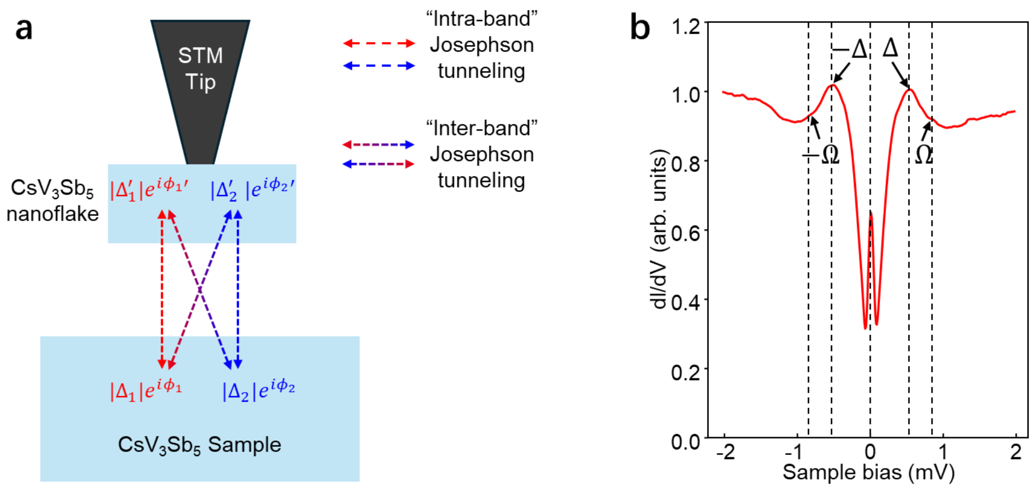

Supplementary Figure 4. Two types of Josephson tunnelling and the signature of Leggett mode. a, Schematic of "intra-band" Josephson tunneling between the same band of sample and tip, and "inter-band" Josephson tunneling between different bands of sample and tip. **b**, The d*I*/d*V* spectrum obtained by the superconducting STM tip, showing the bosonic mode manifest itself at energy ~ Ω (V_s=-10.0 mV, V_{mod}=0.1 mV, I_t=1200 nA).

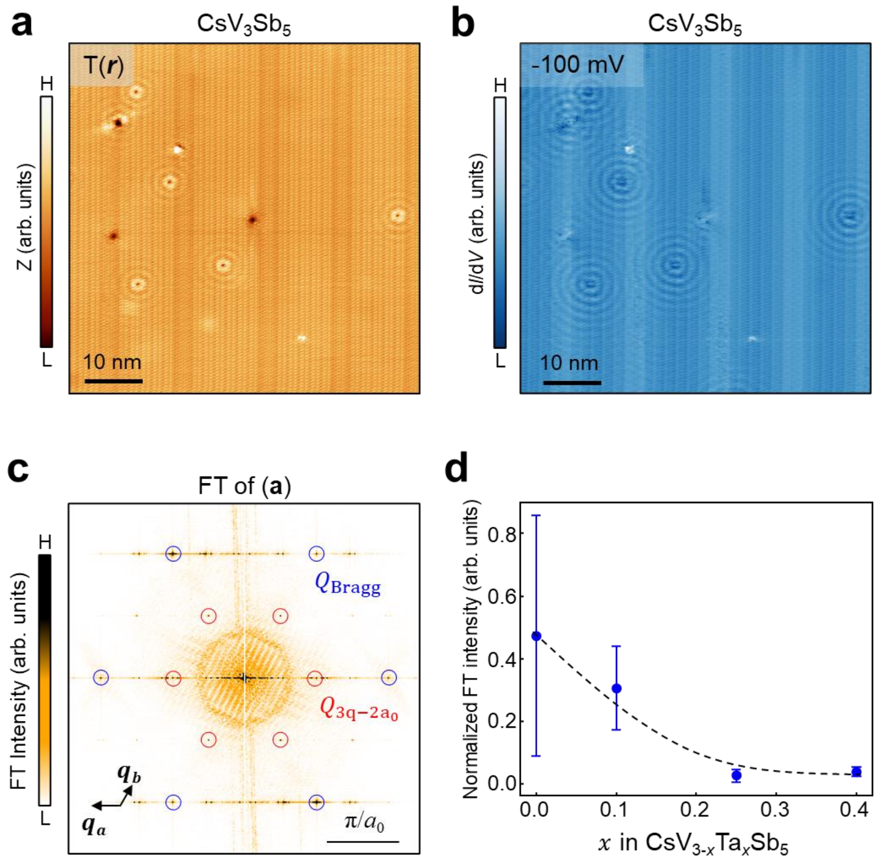

Supplementary Figure 5. Analysis of $2a_0 \times 2a_0$ CDW intensity as a function of Ta-substitution ratio. **a**, STM image of the Sb surface obtained on CsV_3Sb_5 (V_s=-100 mV, I_t=1.5 nA). **b**, Differential conductance map (E=-100 meV) obtained on the Sb surface of CsV_3Sb_5 in the same field of **a** (V_s=-100 mV, I_t=1.5 nA). **c**, Fourier transform (FT) of **a**, showing the Bragg peaks (blue circles) and $2a_0 \times 2a_0$ CDW peaks (red circles), respectively. **d**, The normalized FT intensity extracted from topographies as a function of Ta-substitution ratio x, showing the anti-correlation between x and normalized FT intensity. The normalized FT intensity is defined by $I(Q_{3q-2a_0})/I(Q_{Bragg})$, where a 5×5 pixels window is used to extract the FT intensity in $CsV_{2.75}Ta_{0.25}Sb_5$ and $CsV_{2.60}Ta_{0.40}Sb_5$. The error bars in **d** are determined by the statistical standard deviation along three lattice directions,

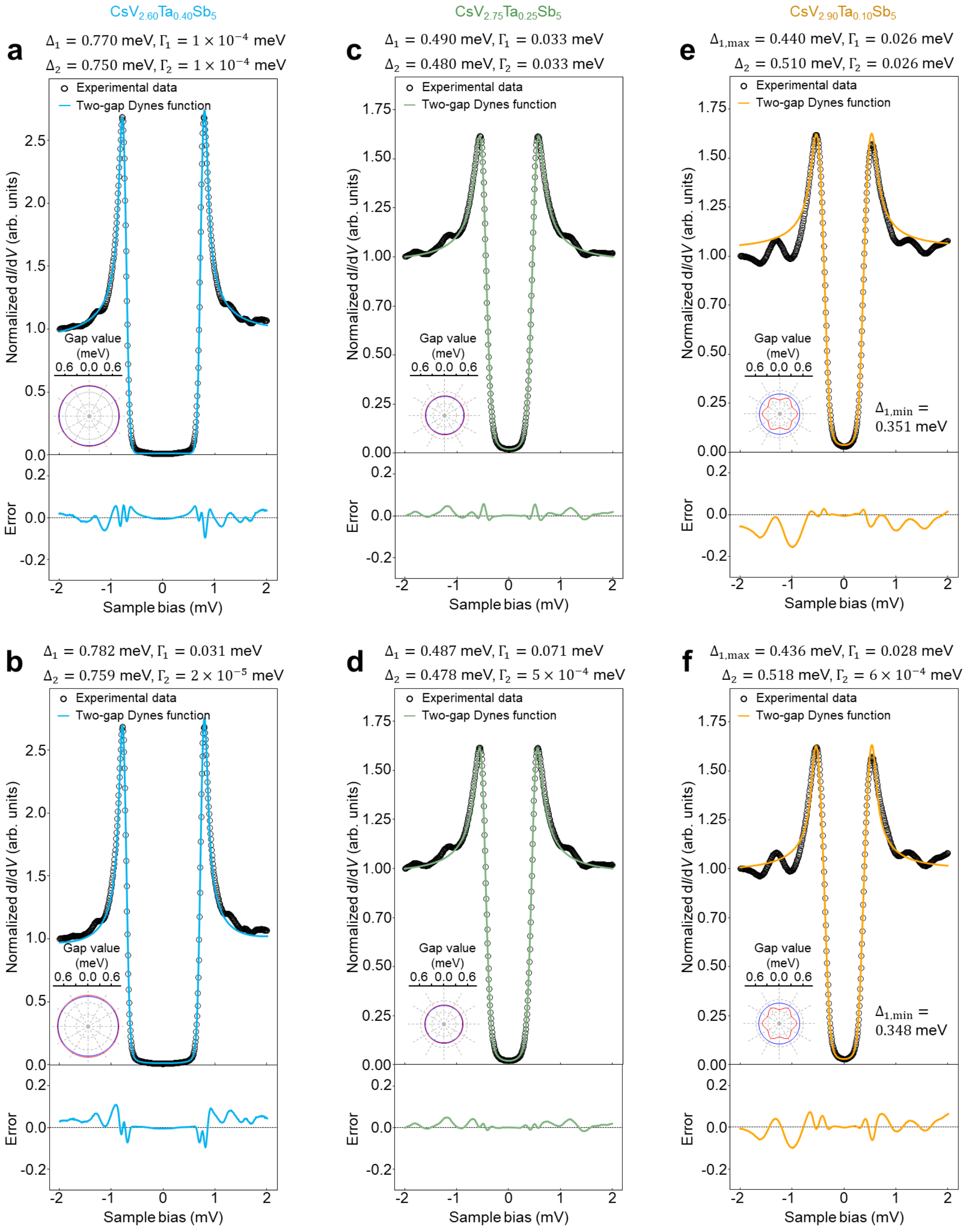

Supplementary Figure 6. Dynes function analysis on the d*I*/d*V* spectra by using the same and the different Γs. a-b, Two-gap Dynes functions description of the spatially-averaged d*I*/d*V* spectra of

$CsV_{2.60}Ta_{0.40}Sb_5$ using the same (a) and different (b) pair-breaking Γs, showing the necessity of including different Γs to capture the superconducting line shape (V_s=-2 mV, V_{mod}=0.05 mV, I_t=1 nA). **c-d**, Two-gap Dynes functions description of the spatially-averaged d*I*/d*V* spectra of $CsV_{2.75}Ta_{0.25}Sb_5$ using the same (**c**) and different (**d**) pair-breaking Γs (V_s=-2 mV, V_{mod}=0.05 mV, I_t=1 nA). **e-f**, Two-gap Dynes functions description of the spatially-averaged d*I*/d*V* spectra of $CsV_{2.90}Ta_{0.10}Sb_5$ using the same (**e**) and different (**f**) pair-breaking Γs (V_s=-2 mV, V_{mod}=0.05 mV, I_t=1 nA).

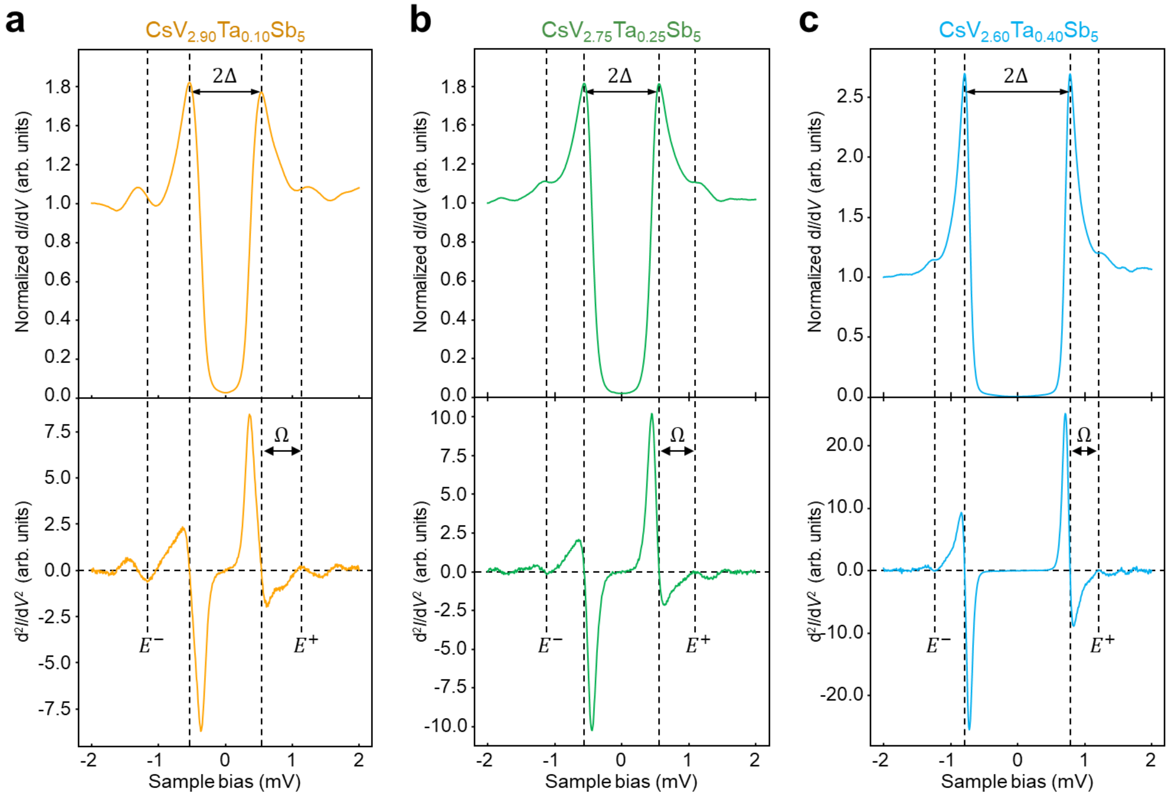

d

	$CsV_{2.90}Ta_{0.10}Sb_5$	$CsV_{2.75}Ta_{0.25}Sb_5$	$CsV_{2.60}Ta_{0.40}Sb_5$
**Δ (meV)**	0.54(0.09)	0.57(0.09)	0.79(0.09)
**Ω (meV)**	0.63(0.13)	0.54(0.13)	0.44(0.13)

Supplementary Figure 7. Analysis and identification of the bosonic mode energy of Ta-substituted $CsV_{3-x}Ta_xSb_5$. a-c, Spatially-averaged d*I*/d*V* (upper panel) and the corresponding derivative spectra d^2I/dV^2 (lower panel) obtained on $CsV_{2.90}Ta_{0.10}Sb_5$ (**a**), $CsV_{2.75}Ta_{0.25}Sb_5$ (**b**), and $CsV_{2.60}Ta_{0.40}Sb_5$ (**c**), respectively (V_s=-2.0 mV, V_{mod}=0.05 mV, I_t=1 nA). **d**, Values of the superconducting peak-to-peak distance Δ and the bosonic mode energy Ω extracted from **a**, **b**, and **c**, respectively. The bosonic mode energy Ω is extracted from $\Omega = (|E^+| + |E^-| - 2\Delta)/2$. The vertical dashed lines in **a**, **b** and **c** present the peaks/dips in d*I*/d*V* and d^2I/dV^2. The horizontal dashed lines present the zero values in d^2I/dV^2.

	CsV_3Sb_5	$CsV_{2.90}Ta_{0.10}Sb_5$	$CsV_{2.75}Ta_{0.25}Sb_5$	$CsV_{2.60}Ta_{0.40}Sb_5$
**a**	0.508	0.961	0.954	0.853
**b**	0.019	0.003	0.000	0.011
c	0.003	0.000	0.000	0.016
α	0.974	0.778	0.500	0.597
$\Delta_{1,max}$(meV)	0.353	0.436	0.487	0.782
$\Delta_{1,\mathrm{min}}$(meV)	0.177	0.348	0.487	0.782
Γ_1(meV)	0.012	0.028	0.071	0.031
Δ_2(meV)	0.629	0.518	0.478	0.758
Γ_2(meV)	3×10^{-4}	6×10^{-4}	5×10^{-4}	2×10^{-5}
A	1.060	0.029	0.018	0.014
σ(meV)	0.004	0.010	0.043	0.011

Supplementary Table 1. Parameters used in the two-gap Dynes functions analysis.
